# Neutralization of Human Cytomegalovirus Entry into Fibroblasts and Epithelial Cells

**DOI:** 10.3390/vaccines5040039

**Published:** 2017-10-31

**Authors:** Felix Wussow, Flavia Chiuppesi, Heidi Contreras, Don J. Diamond

**Affiliations:** Department of Experimental Therapeutics, Beckman Research Institute of the City of Hope, Duarte, CA 91010, USA; fchiuppesi@coh.org (F.C.); hcontreras@coh.org (H.C.); ddiamond@coh.org (D.J.D.)

**Keywords:** cytomegalovirus, neutralizing antibody, fibroblasts, epithelial cells, vaccine, glycoprotein complex, pentamer

## Abstract

Human cytomegalovirus (HCMV) is a leading cause of permanent birth defects, highlighting the need to develop an HCMV vaccine candidate. However, HCMV vaccine development is complicated by the varying capacity of neutralizing antibodies (NAb) to interfere in vitro with the HCMV entry routes mediating infection of fibroblast (FB) and epithelial cells (EC). While HCMV infection of FB and EC requires glycoprotein complexes composed of gB and gH/gL/gO, EC infection depends additionally on the envelope pentamer complex (PC) composed of gH, gL, UL128, UL130 and UL131A. Unlike NAb to gB or gH epitopes that can interfere with both FB and EC infection, NAb targeting predominantly conformational epitopes of the UL128/130/131A subunits are unable to prevent FB entry, though they are highly potent in blocking EC infection. Despite the selective requirement of the PC for EC entry, the PC is exceptionally immunogenic as vaccine antigen to stimulate both EC- and FB-specific NAb responses due to its capacity to elicit NAb that target epitopes of the UL128/130/131A subunits and gH. These findings suggest that the PC could be sufficient in a subunit vaccine formulation to induce robust FB- and EC-specific NAb responses. In this short review, we discuss NAb responses induced through natural infection and vaccination that interfere in vitro with HCMV infection of FB and EC.

## 1. Introduction

Human cytomegalovirus (HCMV) is a leading cause of severe developmental disabilities in newborns and life-threatening illnesses in individuals with a compromised immune system, such as AIDS patients and transplant recipients [1,2]. Based on a cost–benefit analysis by the Institute of Medicine, HCMV vaccine development is considered a “top-tier” national health priority [3]. Yet, despite considerable research efforts of almost half a century, an effective HCMV vaccine candidate remains elusive [4,5,6]. Major impediments in HCMV vaccine development include intricate immune evasion strategies, incomplete protection by natural immunity, establishment of viral latency, unknown immune correlates of protection, and lack of appropriate HCMV animal models [7,8,9,10,11]. Despite these obstacles, encouraging progress in developing an HCMV vaccine candidate has been made with an approach based on envelope glycoprotein B (gB) combined with MF59 adjuvant [12,13]. A Phase II clinical trial with gB/MF59 in women who had given birth within the previous year assessed efficacy rates of 50% to prevent primary HCMV infection [14]. However, a subsequent multicenter trial assessing efficacy of gB/MF59 in HCMV seronegative (HCMV-) adolescents did not reach significance [15]. In addition, a vaccine strategy based on live-attenuated HCMV strain Towne did not show efficacy to prevent primary HCMV infection in mothers whose children attended day care [16]. While a number of vaccine candidates have been shown to ameliorate disease in solid organ and hematopoetic stem cell transplant recipients, gB/MF59 remains the only vaccine that demonstrated partial efficacy to prevent primary HCMV infection in women of childbearing age [4,16,17,18,19,20]. A vaccine formulation that could augment immune responses stimulated by gB alone may therefore provide significant protection against primary maternal HCMV infection, thereby reducing the risk of intrauterine virus transmission.

## 2. Neutralizing Antibody Responses Blocking HCMV Infection of Fibroblast and Epithelial Cells

**Figure 1:**
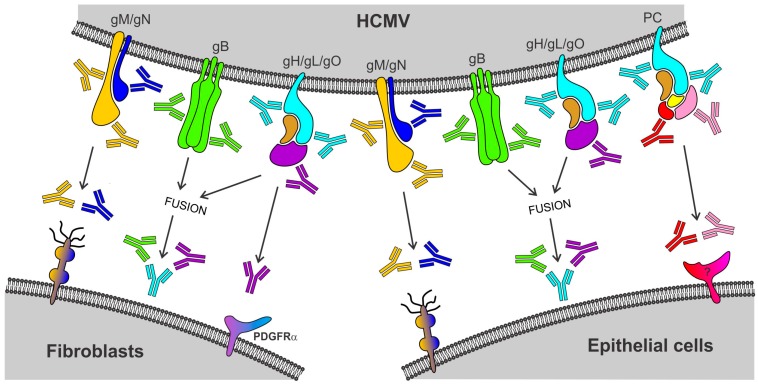
Model for Human cytomegalovirus (HCMV) entry and antibody-mediated neutralization. While HCMV entry into both fibroblasts (FB) and epithelial cells (EC) depends on envelope glycoprotein complexes composed of gM/gN, gB, or gH/gL/gO, HCMV entry into EC additionally requires the pentamer complex (PC) composed of gH, gL, UL128, UL130, and UL131A. While gM/gN may mediate initial attachment via glycosaminoglycans, gB and gH/gL/gO are thought to be involved in fusion of the virion envelope with cellular membranes during entry into both FB and EC, and gH/gL/gO or the PC appears to mediate receptor-binding during entry into FB or EC, respectively. Unlike NAb targeting glycoproteins of gM/gN (yellow and blue antibodies), gB (green antibodies), or gH/gL/gO (cyan and purple antibodies) that can block both FB and EC entry, NAb targeting epitopes of the UL128/130/131A subunits of the PC (red and pink antibodies) are unable to block FB infection, though they are highly potent in blocking EC infection.

**Figure 2:**
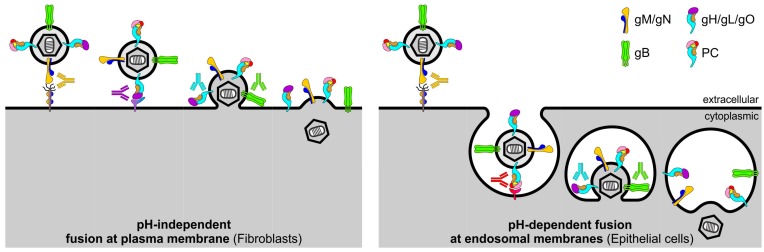
Model of HCMV entry routes and antibody interference. While HCMV entry into FB occurs by fusion at the plasma membrane, HCMV infection of EC is mediated by fusion at the endosomal membrane following endocytosis. During FB and EC entry, initial attachment may be mediated by gM/gN. Receptor-binding during FB entry is thought to occur by interaction of gH/gL/gO with PDGFRα. In contrast, receptor-binding during EC infection is mediated by interaction of the PC with an unknown molecule, which may in turn trigger PC-mediated endocytosis. Upon receptor-binding, gB and gH/gL/gO are thought to be involved in the fusion of the virion envelope with cellular membranes, which may occur either at the plasma membrane during FB entry or at endosomal membranes during EC entry. While NAb targeting gM/gN may interfere with the initial attachment, NAb targeting specific epitopes of gH/gL/gO or the PC may block receptor-binding during FB or EC entry, respectively. In addition, NAb targeting gH/gL/gO and gB may interfere with fusion of the virion envelope and cellular membranes.

Neutralizing antibodies (NAb) that interfere in vitro with glycoprotein complex-mediated virus entry into host cells are thought to contribute to the protection against HCMV infection [21,22,23]. Over the past years it has been recognized that HCMV infection of fibroblasts (FB) and epithelial cells (EC) occurs by distinct routes of entry that depend on an intricate interplay of different sets of envelope glycoprotein complexes. While HCMV entry into FB occurs by pH-independent fusion at the plasma membrane and requires glycoprotein complexes composed of gB and gH/gL/gO, HCMV entry into EC occurs by pH-dependent fusion at the endosomal membrane following endocytosis and depends on gB, gH/gL/gO, and additionally on the envelope pentamer complex (PC) composed of gH, gL, UL128, UL130, and UL131A [24,25,26,27,28,29,30,31] (Figure 1 and Figure 2). Consistent with this HCMV entry model, NAb targeting epitopes of gB and gH can interfere with both FB and EC infection [32,33,34,35]. In contrast, NAb predominantly recognizing conformational epitopes of the UL128/130/131A subunits are unable to block FB infection, though they are substantially more potent than NAb targeting gB or gH epitopes to interfere with EC infection [32,33,35,36]. NAb specific for gO or the gM/gN complex are not well-characterized for their potency to block FB and EC entry, but a few examples of isolated antibodies indicate that NAb targeting these glycoproteins can interfere with both FB and EC infection [32,37]. These in vitro findings suggest that FB-specific NAb responses induced by HCMV are composed of NAb that target epitopes of gB, gH/gL/gO, and gM/gN, whereas EC-specific NAb responses induced by HCMV are composed of NAb specific for the PC in addition to NAb that target gB, gH/gL/gO, and gM/gN (Figure 1).

While our understanding of the glycoprotein complexes that are required for FB and EC infection has increased in recent years, the processes that mediate and neutralize initial attachment, receptor-binding, and membrane fusion during FB and EC infection remain poorly understood [30,38]. Initial attachment during FB entry likely involves the interaction of gM/gN with glycosaminoglycans [39,40,41], but whether this is also the case for EC entry needs to be confirmed [32]. Generally it is well-accepted that gH/gL/gO mediates receptor-binding during FB entry most likely via interaction with platelet-derived growth factor receptor-alpha (PDGFRα) [42,43], whereas the PC mediates receptor-binding during EC entry via a yet unknown molecule (Figure 1 and Figure 2) [44,45]. Other studies suggest an involvement of gB in receptor-binding during FB entry [46,47,48,49], although this remains controversial [27,50]. Following receptor-binding, gH/gL/gO and gB are thought to be involved in fusion of the virion envelope with cellular membranes [24,26,27,28,51,52,53], upon which the nucleocapsid is released into the cell cytoplasm (Figure 2). Membrane fusion may either occur at the plasma membrane during FB infection or at endosomal membranes during EC infection (Figure 2). Based on the HCMV entry routes mediating FB and EC infection it can be hypothesized that (1) gM/gN-specific NAb may block initial attachment during FB and EC entry; (2) NAb specific for gH/gL/gO or the PC prevent receptor-binding during HCMV entry into FB or EC, respectively; and (3) NAb specific for gH/gL/gO and gB may interfere with the fusion of the virion envelope with cellular membranes during both FB and EC infection (Figure 2).

**Figure 3:**
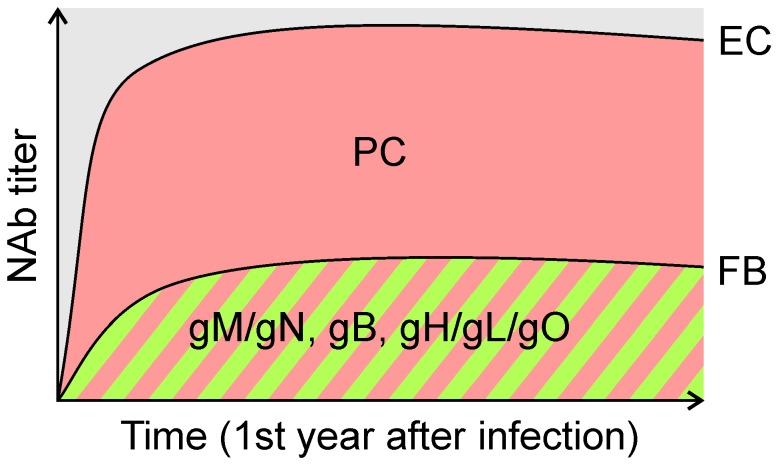
In vitro HCMV NAb responses. During the first year after primary HCMV infection, HCMV+ individuals develop NAb responses with different capacity to prevent in vitro EC and FB infection [56]. NAb measured with EC exceed those measured with FB by orders of magnitude. While FB-specific NAb responses are the result of NAb targeting epitopes of gM/gN, gB, and gH/gL/gO, EC-specific NAb responses are the result of NAb targeting the PC in addition to NAb targeting the gM/gN, gB or gH/gL/gO complexes. The predominant difference in FB- and EC- specific NAb responses is likely a consequence of NAb that target the UL128/130/131A subunits of the PC.

Considering that isolated monoclonal NAb to gB and gH can effectively interfere with both FB and EC infection [32,33,34,35,36,37], antibodies to these envelope glycoproteins may significantly contribute to both the FB- and EC-specific NAb responses measured for HCMV seropositive (HCMV+) individuals in vitro. Similar assumptions may be made for NAb targeting gO or the gM/gN complex based on the potential involvement of these glycoproteins in HCMV entry and the neutralization capacity of isolated NAb targeting these glycoproteins [26,32,37] (Figure 1 and Figure 2). However, hyperimmuneglobulins composed of antibodies of over 1000 HCMV+ individuals contain EC-specific NAb responses that are in majority directed against the HCMV PC [54], suggesting that the PC is the major target of NAb preventing EC infection. Presumably as a result of the exceptional potency of NAb recognizing the UL128/130/131A subunits of the PC, NAb responses of HCMV+ individuals following primary infection are in vitro substantially more potent to interfere with EC infection than with FB infection [55,56] (Figure 3). However, this variable potency of HCMV NAb responses may also be associated with differences in the sensitivity to measure NAb with these cell types possibly as a result of variations in both ligand and receptor copy numbers. Why PC-specific NAb are in vitro substantially more potent to block EC infection than NAb targeting other envelope glycoproteins is unclear, but this could be related to the mechanism of neutralization, antibody affinity, or the low amount of the PC incorporated into virions, which potentially requires only minor amounts of antibody to neutralize PC-dependent entry in vitro [57].

## 3. Vaccine-Mediated Neutralization of HCMV Infection of Fibroblasts and Epithelial Cells

While the gB/MF59 and Towne vaccine candidates can elicit both FB- and EC-specific NAb responses in immunized individuals, they are unable to induce high-titer EC-specific NAb similar to those induced by HCMV during natural infection [55] (Figure 3). These findings may suggest that the limited efficacy of gB/MF59 or the failure of the Towne vaccine to provide significant protection against HCMV infection could be associated with a partial “defect” in mounting robust EC-specific NAb responses. However, other reasons may also account for the deficient vaccine efficacy, including waning antibody titers, insufficient systemic immunity, or the use of a “dead” gB antigen conformation unable to optimally promote HCMV NAb induction [4,6,58]. Several studies converge to the conclusion that the “weak” immunogenicity of gB/MF59 and Towne to elicit EC-specific NAb could be in part related to the inability of these vaccine candidates to elicit anti-PC NAb [59]. As in the case of Towne, HCMV strain AD169 is unable to express a functional PC due to a mutation in the UL128/130/131A gene locus [25,60,61,62]. Restoration of UL128/130/131A gene expression significantly enhances the immunogenicity of HCMV AD169 to stimulate EC-specific NAb responses in mice and rhesus monkeys (RM) [63]. In addition, we and others have demonstrated consistently using different subunit vaccine approaches and animal models that the PC is substantially more immunogenic than gB or gH/gL in stimulating EC-specific NAb responses [35,59,64,65,66,67,68]. Importantly, all five HCMV PC subunits are required to optimally promote the stimulation of EC-specific NAb, indicating that the quaternary conformational neutralizing epitopes within the UL128/130/131A subunits are only formed effectively upon assembly of all five PC subunits [33,64]. These results suggest that the PC could be a major component in a vaccine formulation to stimulate HCMV NAb responses that effectively interfere with EC infection. 

Besides stimulating NAb that potently block HCMV infection of EC, a vaccine approach based on the PC also elicits FB-specific NAb responses that can reach titers of similar or even higher magnitude compared to NAb stimulated by gB or gH/gL complexes [35,59,64]. Consequently, the PC, like gB and gH/gL, has the capacity to elicit NAb responses that block both FB and EC infection, whereby the PC is substantially more immunogenic than gB and gH/gL in eliciting NAb that interfere with EC entry. While the capacity of gB and gH/gL to stimulate NAb that block both FB and EC entry is easy to comprehend, in the case of the PC this may appear contradictory considering the selective requirement of the PC for EC entry and the cell-type specific neutralizing capacity of PC-specific NAb (Figure 1 and Figure 2). Yet, as we and others have shown [33,35], a vaccine approach based on the PC stimulates NAb to epitopes of the UL128/130/131A subunits that potently and specifically interfere with EC entry, and additionally elicits NAb of lower potency to epitopes of gH that interfere with both FB and EC entry (Figure 1). These properties of NAb targeting the UL128/130/131A subunits and gH are consistent with NAb found in HCMV+ individuals and suggest that the difference in EC- and FB-specific NAb responses stimulated by the PC is primarily a result of NAb that target the UL128/130/131A subunits (Figure 3) [32]. Because the PC elicits NAb that target epitopes of the UL128/130/131A subunits and gH [33,35], the PC alone may be sufficient to stimulate HCMV NAb responses that potently interfere in vitro with EC and FB infection. Future studies need to clarify whether a combination of the PC with gB or other envelope glycoproteins could enhance HCMV NAb induction, in particular with regards to FB neutralization and breadth of cross-neutralization activity.

## 4. HCMV Neutralizing Antibodies and Their Potential Protective Capacity In Vivo

Because of the in vitro function of NAb to interfere with HCMV entry, they are considered an important immune component to prevent congenital HCMV infection, although data that support this hypothesis remains relatively sparse [21,22,23]. Considering the proposed glycoprotein complex requirements for the FB and EC pathway of HCMV infection and the neutralization capacity of antibodies targeting the glycoproteins (Figure 1), it can be assumed that NAb targeting epitopes of gB, gH/gL/gO or gM/gN can broadly interfere with HCMV host cell entry and hence potentially contribute significantly to the protection against HCMV infection. In contrast, NAb that specifically interfere with PC-dependent entry may be particularly important to block the infection of specific cell types that are critically involved in HCMV dissemination and horizontal and vertical virus transmission. Based on their in vitro neutralization capacity, PC-specific NAb could potentially play an important role in preventing HCMV acquisition through infection of EC at oral mucosal membranes, cell-associated viremia through endothelial cell infection and virus transfer to leukocytes, or crossing of the fetal-maternal interface through placental cell infection [32,33,35,64,69]. Consistent with these conclusions inferred from in vitro findings for PC-specific NAb, a few studies with clinical samples suggest that NAb targeting the PC may be involved in the control of HCMV spreading in vivo and the prevention of HCMV vertical transmission [21,22].

Since HCMV is highly adapted to humans and unable to replicate efficiently in animals, HCMV vaccine optimization relies on surrogate animal models and their species-specific CMVs, most notably the guinea pig CMV (GpCMV) model of transplacental virus transmission and the rhesus CMV (RhCMV)/RM model [70,71]. While the importance of NAb targeting GpCMV gB or gH in preventing congenital infection is well-documented, the protective capacity of NAb that target the GpCMV homologs of the HCMV UL128/130/131A subunits is unknown [72,73,74,75,76]. Yet, considering that the GpCMV UL128/130/131A homologs are critical for GpCMV pathogenicity and trans-placental virus transmission [77,78], it can be surmised that NAb targeting these GpCMV proteins contribute to the prevention of congenital GpCMV infection. We have shown that vaccine approaches using plasmids or MVA vectors expressing either gB or the PC of RhCMV can reduce plasma viral load in naïve RM following RhCMV challenge [79,80,81,82], indicating that gB- and PC-specific antibodies are involved in the control of RhCMV viremia. In addition, a recent study using a novel non-human primate model of transplacental virus transmission suggests that pre-existing antibodies with high neutralizing activity can protect against congenital RhCMV infection, whereby the reduction in maternal viral load correlated inversely with the rate of transmission [83,84]. These results suggest that a vaccine candidate able to elicit high-titer and sustained NAb could potentially prevent congenital HCMV infection either by significantly reducing maternal viremia or by directly interfering with trans-placental virus transmission. Future studies need to provide a more detailed understating of the protective capacity of NAb targeting the PC or other HCMV envelope glycoprotein complexes using clinical samples and surrogate animal models.

## 5. Conclusions

Although immune correlates of protection against HCMV infection remain unclear, HCMV vaccine development focuses on efforts to enhance the induction of NAb responses as one approach to improve the efficacy rates observed with gB/MF59. In recent years it has been understood that HCMV+ individuals develop NAb responses that in vitro have substantially different capacity to block the HCMV entry routes mediating infection of FB and EC. While NAb targeting the essential envelope glycoprotein complexes composed of gB or gH/gL/gO can effectively interfere with HCMV infection of both FB and EC infection, NAb mainly recognizing conformational epitopes of UL128/130/131A subunits of the PC are exceptionally potent to specifically block EC infection. Because the PC as a vaccine antigen elicits NAb that target epitopes of the UL128/130/131A and gH, the PC stimulates robust NAb that interfere with both EC and FB infection in vitro. These findings suggest the PC could be sufficient in a subunit vaccine formulation to elicit NAb responses that potently and broadly interfere with HCMV host cell entry.
